# Supplementary material for: Empirical methods for controlling false positives and estimating confidence in ChIP-Seq peaks
Source: BMC Bioinformatics. 2008 Dec 5;9:523. doi: 10.1186/1471-2105-9-523 (PMC2628906; doi:10.1186/1471-2105-9-523)
Supplement: Additional File 1 — A variety of html documents from the USeq web site detailing the available applications, their best usage, output file type descriptions, command line menus, etc. [file 1471-2105-9-523-S1.zip › USeqUserGuides/JavaDoc.zip!/JavaDoc/trans/roc/ParseSgrsForParticularRegions.html]

ParseSgrsForParticularRegions


|  |  |  |
| --- | --- | --- |
| **Overview** \| **Package** \| **Class** \| **Use** \| **Tree** \| **Deprecated** \| **Index** \| **Help** | |  |
| **PREV CLASS**   **NEXT CLASS** | **FRAMES**    **NO FRAMES**     **All Classes** |  |
| SUMMARY: NESTED \| FIELD \| CONSTR \| METHOD | DETAIL: FIELD \| CONSTR \| METHOD |  |


---


## trans.roc Class ParseSgrsForParticularRegions

```
java.lang.Object
  trans.roc.ParseSgrsForParticularRegions
```

---

``` public class ParseSgrsForParticularRegions extends java.lang.Object ```

Returns oligo values that overlap particular regions.

---

**Constructor Summary**

`ParseSgrsForParticularRegions(java.lang.String[] args)`


| **Method Summary** | |
| --- | --- |
| `static int` | `inRegion(Sgr sgr, Positive[] pos)`             Checks to see if an sgr object is in one of the positives, if not returns -1, otherwise returns index number. |
| `static void` | `loadRegions(Sgr sgr, Positive[] pos)`             Checks to see if an sgr object is in the positives, if so, adds the sgr object to the positive. |
| `static void` | `main(java.lang.String[] args)` |
| `static Positive[]` | `parseRegionFile(java.io.File picksFile)` |

**Methods inherited from class java.lang.Object**

`equals, getClass, hashCode, notify, notifyAll, toString, wait, wait, wait`

**Constructor Detail**

### ParseSgrsForParticularRegions

```
public ParseSgrsForParticularRegions(java.lang.String[] args)
```


**Method Detail**

### inRegion

```
public static int inRegion(Sgr sgr,
                           Positive[] pos)
```

:   Checks to see if an sgr object is in one of the positives, if not returns -1,
    otherwise returns index number.

---


### loadRegions

```
public static void loadRegions(Sgr sgr,
                               Positive[] pos)
```

:   Checks to see if an sgr object is in the positives, if so, adds the sgr object to the positive.

---


### parseRegionFile

```
public static Positive[] parseRegionFile(java.io.File picksFile)
```

---


### main

```
public static void main(java.lang.String[] args)
```


---


|  |  |  |
| --- | --- | --- |
| **Overview** \| **Package** \| **Class** \| **Use** \| **Tree** \| **Deprecated** \| **Index** \| **Help** | |  |
| **PREV CLASS**   **NEXT CLASS** | **FRAMES**    **NO FRAMES**     **All Classes** |  |
| SUMMARY: NESTED \| FIELD \| CONSTR \| METHOD | DETAIL: FIELD \| CONSTR \| METHOD |  |


---
